# Supplementary figures and images for: Treatment with a VEGFR-2 antibody results in intra-tumor immune modulation and enhances anti-tumor efficacy of PD-L1 blockade in syngeneic murine tumor models
Source: PLoS One. 2022 Jul 18;17(7):e0268244. doi: 10.1371/journal.pone.0268244 (PMC9292077; doi:10.1371/journal.pone.0268244)

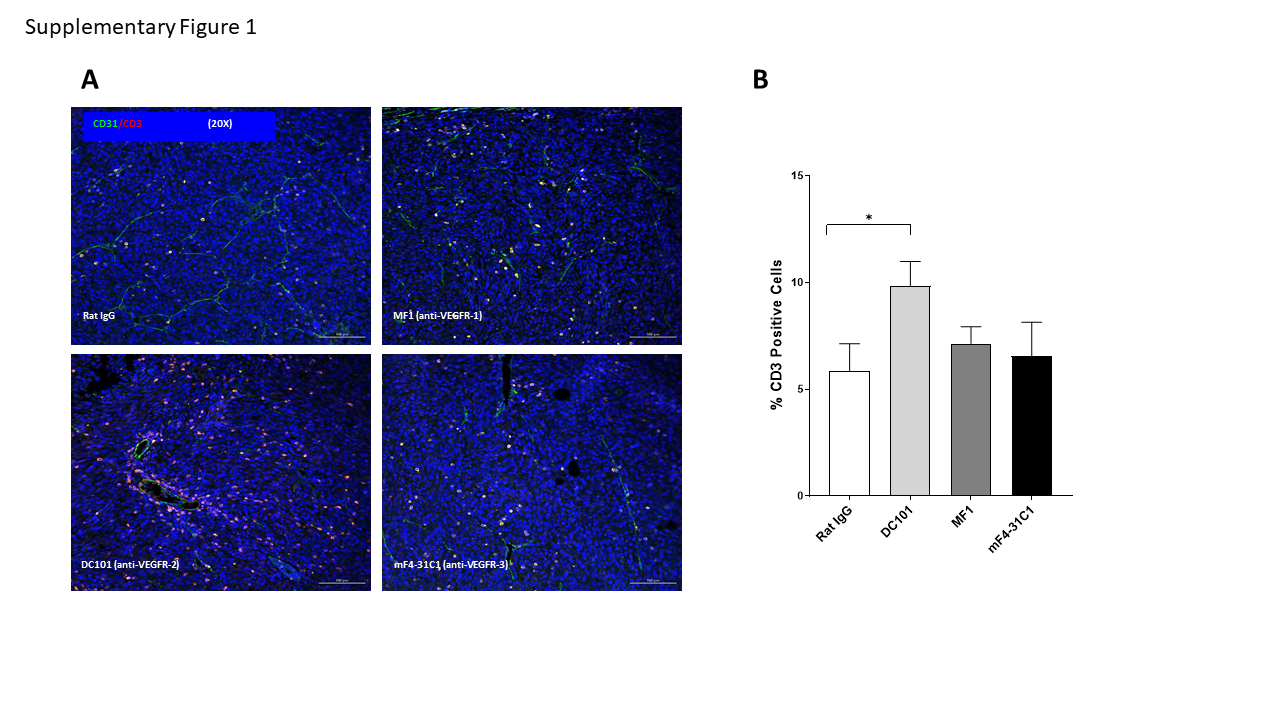

Supplement: S1 Fig — MC38 tumor bearing mice were treated with 40mg/kg Rat IgG, MF1 (anti-VEGFR-1), DC101 (anti-VEGFR-2) and mF4-31C1 (VEGFR-3) at a single dose, and then tumors were collected on day 14. (A) Representative IHC images of CD31 (green), α-smooth muscle actin NG2 (red) and cell nuclei (Hoechst) immunostaining on tumor sections from day 15; (B) Percentage of CD3+ cell infiltrates by IHC (calculated as CD3+ cells over Hoechst positive nuclei) in each tumor sample on day 15 (n = 3 for each group). (TIF) [file pone.0268244.s001.TIF]
